# Engagement of hospital pharmacists and technicians to optimize staffing schedules

**DOI:** 10.1186/s40545-021-00360-5

**Published:** 2021-08-20

**Authors:** Jessica Wright, Richard Arndt, Jason Christensen, Kirstin Kooda, Julie Cunningham

**Affiliations:** 1 Department of Pharmacy Services, Mayo Clinic, Rochester, MN 55905 USA; 2 Department of Pharmacy Services, Mayo Clinic, Eau Claire, WI USA

**Keywords:** Staffing, Schedule, Well-being, Hospital, Restructure, Pharmacist, Technician

## Abstract

Challenges exist in developing work schedules for hospital pharmacy staff due to the need to meet around the clock patient care requirements. Work–life integration and reduced burnout are increasingly important considerations in staff schedules. However, information regarding methods to systematically improve scheduling satisfaction is currently lacking. Hospital pharmacist scheduling surveys were reviewed for solutions in a retreat setting to address growing concerns at our institution. All hospital pharmacists and technicians were surveyed to understand opportunities to improve their schedules. Subsequently, pharmacists participated in a retreat to identify opportunities to share work, prioritize for scheduling improvements, and develop a staffing restructure proposal. Out of 172 pharmacists, 84% completed surveys, whereas 55% of 196 technicians responded. The highest ranked scheduling improvement was a more consistent schedule for both pharmacists and technicians. Several solutions identified during the pharmacist retreat were incorporated into a proposal including decreased weekend staffing frequency (every 3rd to a mix of every 3rd and every 4th), improved scheduling consistency and reduced evenings. Negotiation was among the methods used to identify scheduling solutions. Engagement of frontline staff to lead staffing restructure is expected to ensure success of scheduling changes. Future directions include measuring pharmacist burnout and staff satisfaction before and after change implementation. If successful, the retreat and technician-developed proposal can be used for implementing technician schedule improvements.

## Introduction

Today’s workforce increasingly seeks to maximize work–life integration values [1]. Hospitalized patients require around the clock coverage, presenting challenges to develop schedules that increase satisfaction and reduce burnout. Data suggest greatest dissatisfaction when working evenings and weekends, which is highly reflective of hospital pharmacy staff schedules [2]. Burnout is prevalent among healthcare providers [3, 4], which, in health-system pharmacists, is associated with female gender, distributive roles and longer hours worked per week [5]. Methods to improve pharmacy staff work–life integration and resulting burnout via systematic scheduling improvements are lacking. We describe our approach to address scheduling satisfaction at a single site.

## Methods

Mayo Clinic is a large academic institution with two hospital campuses in Rochester, MN with an integrated pharmacy practice model [6]. Clinically specialized pharmacists provide decentralized patient care from 7 AM to 10:30 PM, 365 days per year. Most pharmacists work a combination of day and evening 8-hour shifts every third weekend, with the exception of overnight pharmacists, who work 10-hour shifts for 7 days followed by 7 days off, alternating with a week of three- or four-day shifts. Pharmacy technicians work a similar model; however, most work every other weekend.

In our institution’s annual survey completed in October 2019, work schedule was voiced as a major contributing factor to burnout among hospital pharmacy staff. Pharmacists and technicians were asked to complete a survey related to staffing in January 2020 and summer of 2020, respectively, and a subsequent pharmacist retreat was held in January 2020 to formulate innovative solutions to the current practice model. The aforementioned surveys were deemed exempt by Mayo Clinic's Institutional Review Board.

## Results

### Pharmacist survey results

Survey completion rate was 144/172 (84%). A majority responded that the highest priority was a consistent schedule; however, 81% favored every 4th weekend staffing in exchange for working longer shifts, more evenings and floating. Floating was expressed as a dissatisfier, but was a lower priority compared with reducing weekend and evening shifts. Ten-hour shifts were preferred over 8- or 12-hour shifts. Overnight pharmacists suggested rotation of holidays as a scheduling improvement but otherwise did not express any other dissatisfiers.

### Technician survey results

Survey completion rate was 108/196 (55.1%). Seventy-six percent responded that they preferred every 3rd weekend staffing over the 13% preferring every other weekend, whereas 11% had no preference. In exchange for working every 3rd weekend, technicians were willing to work longer shifts such as 10 or 12 hours, followed by working more evening shifts, but were least willing to float. Ideal shift lengths were 10 hours, although if working greater than 8 hour shifts, 86% of technicians preferred to have shifts clustered together to allow for longer stretches of time off. A consistent schedule was the highest priority improvement. Changes to the technician staffing model are yet to be evaluated.

### Pharmacist staffing retreat proposal themes

Retreat participants drafted a schedule proposal to develop an every 4th weekend schedule with improved consistency and reduced evenings. Consolidating work shifts was negotiated by leveraging floating, a dissatisfier in the survey. Floating, shared work, swing shifts, and the incorporation of pharmacy resident staffing hours into the weekend model allowed for fewer weekend and evening shifts.

### Pharmacist schedule proposal outcome

Each campus developed separate proposals with planned transitions in late 2021. The smaller campus’s model incorporated the above strategies to reduce weekends for most pharmacists with a mix of every 3rd and 4th weekend. The larger campus incorporated a model of 10-hour evening shifts, 7 days on and 7 days off to reduce overall evening staffing for most pharmacists. Both models resulted in improved consistency of schedules.

## Discussion

Pharmacist and technician surveys revealed similar goals such as a consistent schedule and less frequent weekend shifts and a willingness to exchange for increased shift length. For pharmacists, adding a retreat to the staff survey to develop new pharmacist staffing models engages frontline staff and overcomes survey limitations. Proposal creation empowered frontline staff to determine the optimal strategy for work–life integration. The goal of the hospital pharmacists' staffing structure redesign was to improve pharmacists' work–life integration while better serving patients. Replication of the retreat for technician staffing structure redesign is planned.

Limitations to the staffing proposal methodology exist. The State of Minnesota Board of Pharmacy [7] restrictions around remote order verification, telework and optimal utilization of pharmacy technicians were among barriers that otherwise would have allowed for innovative staffing structures and redistribution of some required functions to pharmacy technicians. Both groups desired 10-hour shifts, but implementing in a model requiring 24/7 patient care coverage results in inefficiencies.

Next steps include validated pharmacist burnout measures pre–post staffing structure implementation and a technician staffing retreat to develop a staffing restructure proposal. COVID-19 pandemic emergency variances lifted restrictions around remote order verification and telework. Maintaining remote work in circumstances such as support roles that complement patient care needs and expanded pharmacy technician activities will further allow for creative solutions in staffing.

## Conclusion

Staff satisfaction regarding work–life integration aids retention and professional growth of staff to help support the highest level of patient care.

## Data Availability

The datasets used and/or analyzed during the current study are available from the corresponding author on reasonable request.

## Abbreviation

COVID-19: Coronavirus disease of 2019
